# Microbial chitinases in the plant holobiont: ecological roles and structural determinants

**DOI:** 10.1093/femsre/fuag022

**Published:** 2026-05-15

**Authors:** Ruben Eichfeld, Alga Zuccaro

**Affiliations:** Institute for Plant Sciences, University of Cologne, 50674 Cologne, Germany; Cluster of Excellence on Plant Sciences (CEPLAS), 50674 Cologne, Germany; Institute for Plant Sciences, University of Cologne, 50674 Cologne, Germany; Cluster of Excellence on Plant Sciences (CEPLAS), 50674 Cologne, Germany

**Keywords:** microbial chitinases, plant microbiota, fungal antagonism, effector protein evolution, plant immune evasion, functional adaptation

## Abstract

Complex intra- and inter-kingdom interactions shape microbiota stability and influence the success of microbial invasion into the plant holobiont. Microbial chitinases, enzymes central to fungal physiology and nutrient acquisition, can also function as secreted effector-like proteins that modulate community dynamics by restricting access to occupied ecological niches, such as plant roots, or by contributing to the destabilization of resident consortia. Beyond these antimicrobial functions, microbial chitinases can facilitate plant colonization by interfering with chitin-triggered immunity, suggesting that chitinase diversification has accompanied microbial adaptation to distinct host-associated niches. In this review, we examine microbial chitinase functions in ecological contexts and discuss how structural variation can tune chitinolytic activity, substrate engagement, and biological function. We argue that the ecological roles of microbial chitinases cannot be inferred from catalytic activity alone, but emerge from the interplay between enzyme architecture, substrate accessibility, synergistic enzyme activities, regulated spatiotemporal expression, and the microbial or host context in which chitin is encountered.

## Teaser

Microbial chitinases act as versatile effector proteins in the plant holobiont, shaping microbiota composition, fungal competition, and plant immune evasion through structural features that tune their activity in distinct ecological contexts.

## Introduction

In plant-associated microbial communities, successful niche establishment depends on diverse intra- and inter-kingdom interactions that shape microbiota stability and plant health. The functional interplay between host plants and their associated microbial communities, collectively referred to as the holobiont, is dynamic and context-dependent (Vandenkoornhuyse et al. 2015, Rosenberg and Zilber-Rosenberg 2016, Hassani et al. 2018). Within this system, microorganisms cooperate, compete, and colonize host tissues to establish niches for proliferation and reproduction. Secreted microbial effector proteins are central to these processes. Bacterial, oomycete, and fungal effectors can manipulate host physiology and immunity, but they can also influence microbiota composition by antagonizing specific microbial taxa (Cook et al. 2015, Lo Presti et al. 2015, Snelders et al. 2020, Eitzen et al. 2021, Eichfeld et al. 2024, Mesny et al. 2024). Thus, niche establishment in the plant holobiont is shaped not only by host-directed effector activities, but also by microbe-directed functions that determine access to occupied ecological space.

As cell walls constitute the initial physical interface between microbial and plant cells, several effector proteins and host enzymes converge on their structural components. In fungi, the cell wall is indispensable for cellular integrity: it counteracts turgor pressure, protects cells against abiotic and biotic stress and provides the surface through which fungi interact with their environment. Yet, the fungal cell wall is not static. Its composition and architecture vary markedly between species and within species in response to environmental conditions, developmental stage, and host interaction (Latgé 2007, Ost et al. 2025). Fungal cell walls are primarily composed of cross-linked homo- and heteropolysaccharides together with structural proteins, which are organized into partially distinct but adaptable layers. A flexible outer layer, enriched in α- or β-1,3/1,6-glucans and glycoproteins, interfaces with the environment, whereas a more rigid inner core is anchored to the plasma membrane and composed mainly of β-glucans that are cross-linked to chitin microfibrils (Latgé 2007). Chitin is a linear polymer of β-1,4-linked N-acetylglucosamine that adopts an antiparallel crystalline arrangement in fungal cell walls, stabilized by hydrogen bonding (Kang et al. 2018, Gow and Lenardon 2023). This crystalline organization renders chitin insoluble and one of the strongest naturally occurring biomaterials, thereby providing a robust structural scaffold within the fungal cell wall. Despite this rigidity, chitin is subject to enzymatic modification that enables fungal growth, development, and adaptation to changing environmental conditions. For example, partial deacetylation by chitin deacetylases reduces crystallinity and increases flexibility of cell wall-embedded chitin (Lindner et al. 2025).

Microbial degradation of chitin polymers is largely mediated by glycoside hydrolases (GHs) of families 18 and 19 (Langner and Göhre 2016, Forsberg et al. 2019). These chitinases hydrolytically cleave β-1,4-glycosidic linkages within chitin chains. However, the extensive cross-linking of chitin to other cell wall polysaccharides and proteins reduces the accessibility for lytic enzymes. Efficient degradation of fungal cell wall material therefore often requires the concerted activity of GHs with distinct substrate specificities, proteases, and other cell wall-active enzymes (Skujins et al. 1965, Lorito et al. 1994, El-Katatny et al. 2001, Benítez et al. 2004, Gruber and Seidl-Seiboth 2012). In addition, the crystalline structure of chitin can restrict access by hydrolytic chitinases. Microbial lytic polysaccharide monooxygenases (LPMOs) of auxiliary activity families 10 and 11 (AA10, AA11) can assist the degradation of crystalline chitin by using an oxidative reaction mechanism to introduce strand breaks into chitin fibers, generating oxidized chain ends (Vaaje-Kolstad et al. 2010, Hemsworth et al. 2014). These strand breaks can increase the number of accessible sites for chitinases, which then continue hydrolytic degradation (Vaaje-Kolstad et al. 2013). The concerted action of LPMOs and chitinases has also been implicated in fungal cell wall remodeling (Yao et al. 2023). In addition to enzyme synergy, substrate engagement can be enhanced by remote substrate-binding structures present in some chitinases, which increase proximity between the catalytic domain and insoluble chitin. Thus, efficient chitin degradation in fungal cell walls depends not only on catalytic activity, but also on synergistic enzyme systems and structural features that promote access to complex, crystalline substrates.

Chitinases are distributed across all kingdoms of life and fulfill diverse functions within plant-associated microbial communities. Plants employ chitinases to restrict fungal invasion, whereas bacteria can use chitinases to mobilize carbon and nitrogen from fungal cell walls and to antagonize competing fungi during niche establishment. Fungi themselves require chitinases for fundamental physiological processes, including cell wall remodeling during growth and development, but can also deploy them during competitive interactions and nutrient acquisition in complex microbial environments.

In this review, we examine evidence that microbial chitinases can act as effector-like proteins in diverse biotic and ecological contexts, ranging from fungal antagonism to host plant colonization. Through these activities, microbial chitinases can contribute to microbial community stability and, consequently, to the maintenance of a functional plant holobiont. We argue that the biological roles of chitinases cannot be inferred from catalytic activity alone. Instead, they emerge from the interplay among enzyme architecture, substrate accessibility, synergistic enzyme activities, regulated expression, and ecological context. Linking structural variation within and beyond the catalytic center to specific microbial and host-associated environments is therefore essential for understanding the ecological functions of chitinolytic enzymes.

## Microbial chitinases in community stability and microbial invasion

A stable plant-associated microbiota can function as an extended immune barrier against microbial invaders (Mendes et al. 2011, Durán et al. 2018, Sarkar et al. 2019, Mesny et al. 2024). This barrier is not only the result of antagonistic interactions between individual microbiota members and incoming microbes but also depends on synergistic intra- and inter-kingdom interactions among resident taxa (Mahdi et al. 2022). Host plants can further shape these protective functions by recruiting specific microbes in response to pathogenic or non-pathogenic immune-stimulating organisms, a process often referred to as a cry-for-help response (Berendsen et al. 2012, Rolfe et al. 2019, Liu et al. 2024, Elad et al. 2025). Although microbial cell wall-degrading enzymes (MCWDEs) and secondary metabolites contribute to the suppression of invading microbes, the mechanisms underlying microbiota-mediated pathogen inhibition remain incompletely understood. Here, we propose that bacterial and fungal chitinases represent a functional component of stable, plant-protective microbial communities. Conversely, whether phytopathogen-secreted chitinases contribute to the destabilization of multi-kingdom plant microbiota remains an important and testable hypothesis.

### Bacterial chitinases contribute to pathogen inhibition in disease-suppressive soils

Bacterial chitinases belong to GH families 18 and 19. Over recent decades, multiple GH18 and GH19 chitinases derived from bacterial model organisms and soil samples have been shown to inhibit fungal plant pathogens and reduce plant disease symptoms (Shapira 1989, Neeraja et al. 2010, Hjort et al. 2014). In addition, chitinolytic activity in amended soil correlates positively with suppression of *Verticillium* wilt, suggesting that chitin-degrading members of the resident microbiota can contribute to fungal disease suppression (Inderbitzin et al. 2018). Particularly compelling evidence for a role of bacterial chitinases in plant-protective microbiota functions comes from the root-endophytic microbial community of sugar beet (Carrión et al. 2019). Upon infection with the filamentous fungal pathogen *Rhizoctonia solani*, sugar beet recruits Proteobacteria and Bacteroidetes enriched in biosynthetic gene clusters (BGCs) and fungal cell wall-degrading enzymes (FCWDEs). Among members of the Chitinophagaceae, Burkholderiaceae, and Xanthomonadaceae, chitinases represent a prominent class of enriched FCWDEs. Isolation and re-introduction of these chitinolytic taxa into the diseased system revealed strong transcriptional induction and enzymatic activity of the identified chitinases, together with a disease-suppressive effect. This protective activity was further supported by strains expressing nonribosomal peptide synthases (NRPs) and polyketide synthases (PKSs), indicating that chitinases likely act as part of a broader antimicrobial repertoire that includes antifungal secondary metabolites (Carrión et al. 2019).

**Figure 1:**
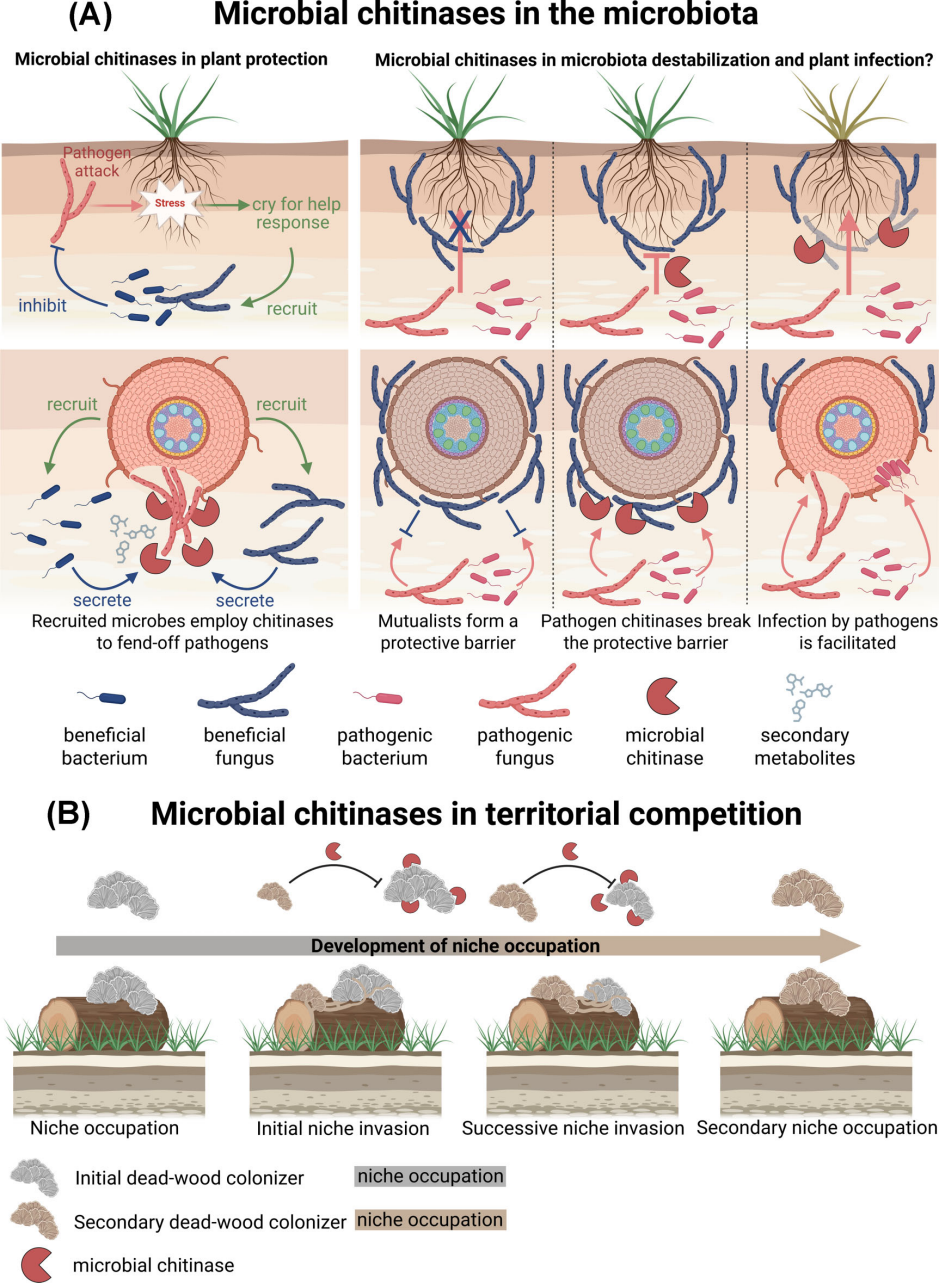
Microbial chitinases display antifungal activities for different ecological purposes. A) Microbial chitinases in community contexts. Chitinases derived from plant-beneficial fungi and bacteria can prevent ingress of fungal invaders into resident stable communities by degrading chitin embedded in fungal cell walls. Infected plants can actively recruit fungi and bacteria with high chitinolytic potential upon pathogen attack in a cry-for-help response. Microbial-derived chitinases act in concert with other antimicrobial effector proteins and secondary metabolites during pathogen restriction. Whether pathogenic fungi and bacteria in turn employ chitinases to de-stabilize plant-protective microbial communities by targeting beneficial fungi is currently unknown but discussed e.g. for the bacterial pathogen Xylella fastidiosa (Landa et al. 2022). B) Microbial chitinases in territorial competition. Especially in saprotrophic Basidiomycetes, chitinases have important functions in territorial competition and niche invasion. For example, several wood-decaying fungi, such as Phanerochaete chrysosporium express and secrete a cocktail of chitinases to, in concert with other antimicrobials, fend-off niche competitors. By degrading the chitinous biomatter of the competitors, the niche can be re-colonized by the secondary colonizer. In addition, carbon and nitrogen are being mobilized and fed into local food webs (Karlsson et al. 2016, Maillard et al. 2023). Figure was created using Biorender.

Although the study by Carrión et al. did not generate chitinase deletion strains or test recombinant chitinases, the study highlights chitinases, together with other FCWDEs and secondary metabolites, as candidate contributors to a stable, protective microbiome. It further suggests that chitinase activity is embedded in a broader communication network involving plant-mediated recruitment of chitinolytic bacteria and synergistic interactions among microbial community members. Thus, bacteria appear to combine multiple antimicrobial functions, including chitinolytic activity, to compete within microbial communities and support host protection, thereby contributing to plant holobiont stability (Fig. 1A). However, FCWDEs, including chitinases, only partially explain the protective functions of the microbiota. How these hydrolytic enzymes act in concert with other microbial effectors and metabolites remains an important question.

If bacterial chitinases contribute to niche defense against pathogenic fungal invaders, they may also act in other ecological directions. One possibility is that pathogen-associated chitinases target fungal members of the resident microbiota and thereby contribute to community destabilization during host infection (Fig. 1A). Such a role remains speculative but is conceptually consistent with the broader observation that antimicrobial effector proteins can remodel resident microbiota and support plant colonization by pathogens (Chang et al. 2021, Snelders et al. 2021, Gómez-Pérez et al. 2023, Ökmen et al. 2023, Snelders et al. 2023). In the bacterial pathogen *Xylella fastidiosa*, infection is accompanied by pronounced compositional shifts in the plant-associated microbiota, which have been hypothesized to be partly mediated by the bacterium’s chitinolytic machinery, including the GH18 chitinase ChiA (Landa et al. 2022). Notably, *X. fastidiosa* ChiA deletion mutants show strongly reduced disease development in grapevines (Labroussaa et al. 2017). Although this ChiA-dependent disease phenotype has not been directly linked to microbiota modulation, comparative profiling of the mycobiota in plants infected with wild-type and *ChiA*-deficient *X. fastidiosa* could test whether ChiA activity affects specific fungal taxa during disease development.

### Fungal chitinases contribute to antagonism and niche establishment

Among fungi, chitinases are ubiquitously distributed and are so far known to belong exclusively to the GH18 family. The number of chitinase-encoding genes varies widely across species, with generally higher copy numbers in the genomes of filamentous fungi (Langner and Göhre 2016, Goughenour et al. 2021). Several chitinases from filamentous fungi have been implicated in fungal antagonism and mycoparasitic interactions. Prominent examples are found among Ascomycetes, particularly species of the genus *Trichoderma. Trichoderma* spp. display diverse lifestyles, ranging from saprotrophy to endophytism, and occur as natural members of the plant microbiota (Harman et al. 2004, Guzmán-Guzmán et al. 2019, Scott et al. 2023, Woo et al. 2023). In these contexts, they can protect host plants from various fungal pathogens (Benítez et al. 2004, Elad et al. 2025). For example, the *T. harzianum* chitinases ECH42 and Chit33 have been implicated in antagonism against plant pathogens such as *R. solani* and *Botrytis cinerea* (Limón et al. 1999, Woo et al. 1999, Zeilinger et al. 1999), supporting the view that fungal chitinases can contribute to host-protective microbial interactions (Fig. 1A). Importantly, chitinases do not act in isolation during fungal antagonism. Competitive and mycoparasitic fungi, including *Trichoderma* species, secrete enzyme cocktails during fungal encounters that include chitinases, glucanases, and other FCWDEs (Djonović et al. 2006, Druzhinina et al. 2011). Such coordinated hydrolytic activity likely enhances access to, and degradation of, the complex fungal cell wall.

Although most well-characterized fungal chitinases come from Ascomycetes, Basidiomycetes also engage in territorial competition (Boddy 2000, Lindahl et al. 2001) and appear to employ chitinases in this context. For example, the white-rot fungus *Phanerochaete chrysosporium* overgrows initial colonizers of decaying wood and induces a distinct set of chitinases during niche invasion. Expression of these chitinases is stronger during encounters with living competitors than with dead mycelium, suggesting that they are not only involved in nitrogen or carbohydrate mobilization, but may also contribute to active territorial competition (Karlsson et al. 2016). Similar observations have been reported for *Coniophora arida, Hypholoma capnoides*, and *Resinicium bicolor* (Lindahl and Finlay 2006), indicating that chitinase induction during fungal encounters may be a broader feature of Basidiomycete niche occupation (Fig. 1B).

Functional evidence for this role has recently emerged from the beneficial root endophytes *Serendipita indica* and *Serendipita vermifera*. These fungi specifically express orthologous GH18-CBM5 chitinases, *Si*CHIT and *Sv*CHIT, during confrontation with the hemibiotrophic soil-borne plant pathogen *Bipolaris sorokiniana*. Both chitinases suppress pathogen growth prior to root infection, supporting a role in niche defense and host protection (Eichfeld et al. 2024). In parallel, degradation of fungal biomass can mobilize carbon and nitrogen from chitin, thereby contributing to nutrient cycling, particularly in wood-decaying ecosystems where Basidiomycetes with high chitinolytic potential are abundant (Maillard et al. 2023). Together, these studies suggest that GH18 chitinases contribute to Basidiomycete territorial competition while also linking fungal antagonism to nutrient acquisition.

Although fungal chitinases can support niche establishment and protection within specific ecological settings, clear functional evidence for broad microbiota manipulation by fungal chitinases, comparable to observations for chitinolytic bacteria, is still lacking (Carrión et al. 2019). Plant-protective fungi such as *T. harzianum* can be recruited during cry-for-help responses (Lombardi et al. 2018), but whether their chitinases directly contribute to community-level microbiota restructuring remains unresolved. Conversely, fungal pathogens may also use chitinases to target fungal members of the host-associated mycobiota and thereby facilitate disease establishment (Fig. 1A). This hypothesis is conceptually supported by the fungal effector AMP3 from *V. dahliae*, which targets fungi in the plant endosphere and promotes host infection (Snelders et al. 2021). However, direct evidence for an analogous role of pathogen-secreted chitinases in mycobiota destabilization is currently missing.

## Structural variation shapes context-dependent chitinase functions

Microbial chitinases with antifungal ability act on chitin within fungal cell walls, thereby compromising cell wall stability and cellular integrity. Catalytic activity is a prerequisite for this antifungal function (Eichfeld et al. 2024), but it does not alone explain why different chitinases act efficiently on different substrates or in different ecological contexts. Structural features within the catalytic domain can strongly influence substrate preference and product formation. For example, the depth and architecture of the catalytic cleft affect substrate engagement, with longer and deeper clefts often supporting activity on insoluble and crystalline chitin fibers (Eijsink et al. 2008). In addition, variation in single aromatic amino acids can shift chitinases between processive and non-processive modes of substrate degradation, resulting in different substrate preferences and product profiles (Horn et al. 2006, Zakariassen et al. 2009, Hoell et al. 2010). Amino acid variation in substrate- or product sites has also been associated with adaptive diversification of *Trichoderma* chitinases, potentially reflecting selective pressures imposed by microbial confrontation and mycoparasitism (Ihrmark et al. 2010). Beyond the catalytic center, remote structural elements can further modulate substrate binding, enzyme accessibility, and biological activity. However, ecological function is not determined by enzyme structure alone, but also by regulated expression in response to microbial competitors, host colonization, or environmental cues. We therefore propose that context-dependent chitinase functions, including fungal antagonism and host immune evasion, emerge from the interplay between structural variation, substrate availability, and spatiotemporal deployment.

### Substrate-binding motifs and remote loops: functional adaptations of chitinases in microbial competition

In addition to a catalytic GH domain, bacterial and fungal chitinases often contain one or more carbohydrate-binding modules (CBMs) which are attached to the N- or C-terminus of the catalytic GH domain through flexible linker regions. Chitinase-associated CBMs mainly belong to the families 1, 5, 12, 14, 18, 50, and 73. CBM5, CBM12, and CBM73 are predominantly found in bacteria, CBM1, CBM18, and CBM50 are common in fungi, whereas CBM14 is broadly distributed across domains of life. Notably, GH18-associated CBM5 domains occur in fungi primarily in Agaricomycetes and a few Mucoromycetes but are absent from Ascomycetes (Eichfeld et al. 2024). CBMs are small, non-catalytic domains, typically 40–70 amino acids in length, that facilitate substrate binding through aromatic residue-mediated stacking interactions and thereby enhance chitin degradation (Watanabe et al. 1994, Kezuka et al. 2006, Uni et al. 2012, Stockinger et al. 2015, Liu et al. 2023b). Importantly, CBMs can support chitin degradation not only in simple enzyme-substrate systems but also in the presence of non-substrate polysaccharides (Liu et al. 2023b), where they may help to maintain efficient and specific catalysis in complex natural environments such as fungal cell walls.

Different CBM families mediate distinct binding specificities toward substrates and substrate surfaces. CBM1 domains can bind both cellulose and chitin (Limón et al. 2001), whereas CBM50 domains bind small oligomeric chitooligosaccharides (COS) (de Jonge et al. 2010). CBM73 displays strong affinity towards polymeric chitin but not COS (Forsberg et al. 2016, Madland et al. 2021). CBM12 increases binding and activity on crystalline and colloidal chitin, whereas the closely related CBM5 preferentially binds to crystalline chitin (Liu et al. 2023b). The positive effect of CBM5 on binding and degradation is most evident for insoluble chitin but not for soluble COS (Hashimoto et al. 2000, Limón et al. 2001), suggesting that efficient CBM5-substrate interactions depend on higher-order substrate architecture. Consistent with this view, CBM5 domains can enhance degradation of insoluble chitin, while reducing activity on soluble COS (Itoh et al. 2006, Uni et al. 2012). This points to a functional trade-off between efficient engagement of insoluble, structured substrates, and turnover of small and soluble oligomers. Thus, CBMs can tune the functional capacity of bacterial and fungal chitinases by influencing substrate targeting, degradation efficiency, and activity in complex cell wall environments. In ecological contexts, these properties may increase the ability of chitinases to attack fungal cell walls during direct microbial competition or within complex multi-kingdom communities.

**Figure 2:**
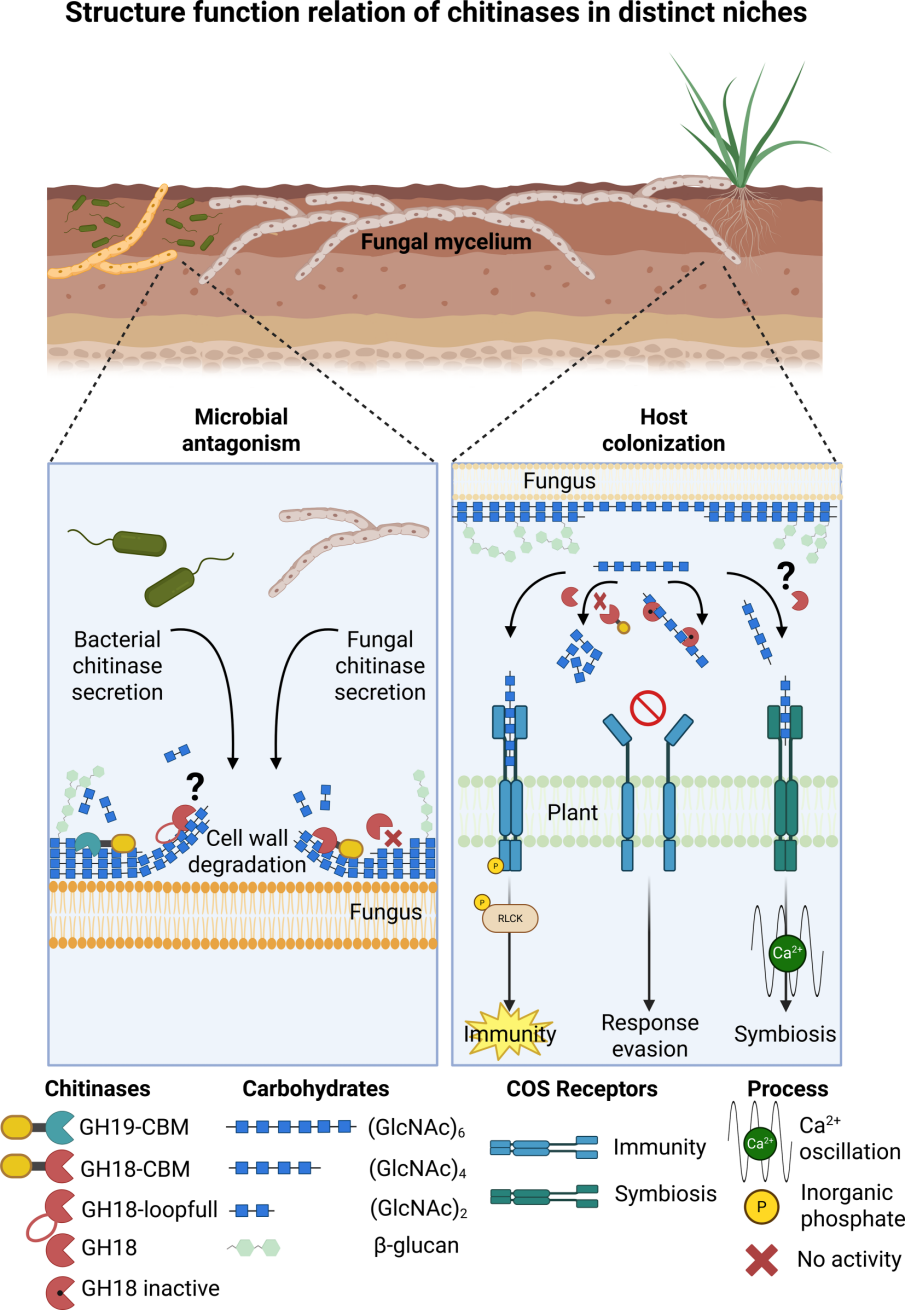
Microbial chitinases display distinct ecological functions depending on structural variations. During fungal antagonism, fungal and bacterial chitinases benefit from additional substrate-binding motifs, such as CBMs. CBMs are found attached to the catalytic GH18 or GH19 domains at the N- or C-terminus and facilitate the degradation of crystalline chitin that is embedded in complex structural scaffolds, such as fungal cell walls. Removal of CBMs can lead to a loss of antifungal ability, while basal chitinolytic activity is retained. In turn, fusion of CBMs to GH18 or GH19 domains can activate their antifungal potential. Some plant-derived chitinases bear remote loops that render these chitinases antifungal by facilitating substrate binding. Whether such loops have similar functions in microbial-derived chitinases is currently not known. During host colonization, fungal-derived chitooligosaccharides (COS), such as chitohexaose, can be recognized by immune receptor pairs, leading to downstream immune signaling. Fungal chitinases can function as effectors by degrading immunogenic COS into smaller fragments, such as chitobiose. Catalytically inactive chitinase-like effectors can sequester immunogenic COS, thereby hiding them from recognition by host immune receptors. COS can also serve as signals in symbiosis signaling, but whether chitinases are involved in the production of these symbiotic signals remains unknown. Figure was created using Biorender.

CBM5 domains have been studied particularly for their contribution to the antifungal activity of microbial chitinases. In bacterial chitinases, such as ChiC from *Streptomyces griseus*, or ChiB from *Nocardiopsis prasina*, removal of the CBM5 domain or mutation of aromatic tryptophan residues required for substrate binding, abolishes inhibition of *T. reesei* hyphal extension (Itoh et al. 2002, 2006, Tsujibo et al. 2003). In fungi, GH18 chitinases coupled to CBM5 domains are expressed during inter-fungal confrontation in different species (Karlsson et al. 2016, Eichfeld et al. 2024) and removal of CBM5 from fungal GH18 chitinases decreases antifungal activity against phytopathogenic fungi (Eichfeld et al. 2026). Together, these studies support a conserved contribution of CBM5 domains to the antifungal potential of microbial chitinases, most likely by enhancing engagement with insoluble chitin in fungal cell walls (Fig. 2).

In contrast, the contribution of other CBM families to antifungal chitinase functions has been assessed in only a few cases. For example, *T. harzianum* strains overexpressing ECH42 or Chit33 artificially fused to the CBM1 of a cellobiohydrolase showed increased capacity to overgrow the fungal pathogens *R. solani, B. cinerea*, and *Phytophthora citrophthora* (Limón et al. 2004). Although *T. harzianum* and other mycoparasitic *Trichoderma* species encode numerous chitinases that naturally contain CBM1, CBM18, or CBM50 domains, the effects of domain truncation or exchange on chitin degradation and antifungal activity remain largely unexplored. A systematic comparison of how different CBM families contribute to chitinase activity and biological function is therefore still missing. Such analyses could be achieved by constructing chimeric chitinases in which the catalytic GH18 domain is retained, while different CBMs are fused to the N- or C-terminus.

It also remains unclear whether additional, less obvious structural elements contribute to the antifungal activity of bacterial or fungal chitinases. An informative example comes from GH19 chitinases of the moss *Bryum coronatum*. These chitinases differ in loop structures and the presence or absence of these loops affect their activity spectra: loop-containing chitinases display stronger activity on polymeric chitin substrates and antifungal activity, whereas loopless variants are less active on polymeric chitin and are inactive against fungal pathogens, but degrade short COS more efficiently than their loop-containing analogs (Taira et al. 2011). More recently, these loops were shown to enhance binding to chitin in the fungal cell wall, linking this structural feature to both chitin degradation and antifungal activity (Kozome et al. 2024). Whether analogous loop structures occur in bacterial or fungal chitinases, and whether they have comparable effects on substrate binding or antifungal activity, remains unknown. Addressing this question would broaden our understanding of how structural elements outside the catalytic center contribute to chitinase function in ecological contexts (Fig. 2).

The trade-off between degradation of insoluble and soluble substrates seems to have different structural determinants. On the one hand, the presence of remote structures that mediate binding, including loops, as in *B. coronatum* (Taira et al. 2011), or CBMs, such as in *S. griseus* or *N. prasina* (Itoh et al. 2006, Uni et al. 2012) can increase binding efficiency towards crystalline substrates, while limiting catalytic efficiency on soluble oligosaccharide substrates or at substrate saturation possibly via limiting access to the catalytic cleft, thereby reducing association rates, or hindering release, causing reduced dissociation rates (Itoh et al. 2006, Uni et al. 2012, Sørensen et al. 2015). This functional relation seems to depend on the chitinase and the kind of soluble substrate as well, as other studies do report this trend with soluble chitin but not chitooligosaccharides (Katouno et al. 2004). Variation in these remote elements could therefore determine biological functions of chitinases independent of the catalytic core. On the other hand, point mutations of aromatic residues in the binding groove of the catalytic cleft can cause reduced enzyme processivity and therefore decreased reaction velocity on crystalline substrates. In turn, this loss of processivity accelerates degradation of soluble chitin oligosaccharides (Watanabe et al. 2003, Horn et al. 2006, Zakariassen et al. 2009, 2010). This shows that variation in remote structures but also single aromatic residues in the catalytic cleft can cause nuanced substrate-dependent activity shifts. In the next chapter, we discuss that such variation in remote structural elements and the catalytic cleft may offer a source for functional specialization of chitinases in diverse biotic interactions and ecological contexts.

### Fungal chitinolytic enzymes can serve as effectors in host-immune evasion

During host colonization, plant chitinases can partially degrade fungal cell walls and release soluble COS. These COS are sensed by plant transmembrane receptor-like kinases carrying extracellular CBM50/LysM modules (LysM-RLKs). COS binding to LysM-RLKs promotes receptor complex formation with the co-receptor CERK1, which is conserved across plant species. Following COS-induced receptor complex formation, the intracellular kinase domain of CERK1 undergoes autophosphorylation and trans-phosphorylates downstream immune signaling components, including receptor-like cytoplasmic kinases (RLCKs). This signaling cascade activates reactive oxygen species (ROS) production, cytosolic calcium influx, and defense gene expression (Gong et al. 2020).

To colonize host plants, fungal pathogens and mutualists have evolved strategies to interfere with COS-induced receptor activation and downstream immune signaling. These strategies include effector proteins that target cytosolic immune signaling components (Bai et al. 2019, Irieda et al. 2019), chitin deacetylases that convert chitin oligomers into less immunogenic, partially de-acetylated oligomers (Cord-Landwehr et al. 2016, Gao et al. 2019), and fungal effectors that inhibit plant chitinases to prevent the release of immunogenic COS (Jashni et al. 2015, Ökmen et al. 2018). In addition, small CBM-containing effectors can shield immunogenic chitin fragments from recognition. In the pathogen *Cladosporium fulvum*, for example, the CBM50/LysM-containing effector Ecp6 sequesters free COS with higher affinity than plant LysM-RLKs (de Jonge et al. 2010), whereas the CBM14-containing effector Avr4 protects chitin in the fungal cell wall from enzymatic degradation (van den Burg et al. 2006). Thus, fungal manipulation of chitin availability, structure or perception is a well-established mechanism of host immune evasion. By contrast, the contribution of fungal chitinolytic enzymes to this process has only recently begun to emerge.

Chitin-induced immune activation depends on the degree of polymerization (DP) and acetylation pattern of COS released from fungal cell walls into the apoplast. Fully acetylated COS with a DP ≥ 6 can induce immune responses, whereas COS with a DP < 6 generally do not (Cao et al. 2014, Gubaeva et al. 2018). However, fully acetylated COS with a DP > 6 have limited solubility in aqueous solution and may therefore not be readily released from chitin fibers even after chitinase activity. This raises the question of which chitin-derived fragments are naturally available for perception by plant immune receptors during fungal colonization. Fungal cell walls contain regions of partially de-acetylated chitin with the degree and pattern of acetylation varying between species and developmental stages (Urs et al. 2023). Plant chitinases may therefore release partially de-acetylated COS (paCOS) which are more soluble under apoplastic conditions. Although strongly de-acetylated fragments may lose immune activity, paCOS with a high degree of acetylation can still function as elicitors (Cord-Landwehr et al. 2016, Gubaeva et al. 2018, Cord-Landwehr et al. 2020). In addition, longer oligomers containing several de-acetylated units but block-wise acetylated stretches can activate immunity through the same receptor systems (dos Santos et al. 2008, Richter et al. 2025) Thus, degradation of immunogenic COS and paCOS with DP ≥ 6 represents a plausible mechanism by which fungal enzymes can reduce chitin-triggered immune activation.

Consistent with this idea, the powdery mildew fungus *Podosphaera xanthii* secretes a suite of small effectors with chitinase activity (EWCAs) at the penetration site during melon infection. EWCAs do not contain the DxxDxDxE motif characteristic of GH18 chitinases but are structurally related to AA10 LPMOs and contain predicted binding sites for chitin and copper ions. These proteins degrade soluble, immunogenic COS into shorter, non-immunogenic COS with DPs of 2–5, thereby preventing LysM-RLK/CERK1 receptor dimerization, evading chitin-triggered immunity and contributing to host infection (Martinez-Cruz et al. 2021). Notably, EWCAs act on soluble COS but not on insoluble, crystalline chitin embedded in fungal cell walls, suggesting specialization toward immune suppression rather than cell wall degradation. A similar preference for soluble oligomeric substrates accompanied by reduced activity on crystalline substrates has been observed for some fungal AA11 LPMOs (Rieder et al. 2021), although the structural determinants underlying this substrate preference remain unresolved. Because homologs of EWCAs occur in diverse fungal pathogens, including Ascomycetes and Basidiomycetes, this mechanism may be more broadly distributed among fungal invaders.

LPMO-mediated cleavage typically generates oxidized products, raising the possibility that oxidized chitin fragments may have distinct biological activities. During infection by *Botrytis cinerea*, fungal AA9 LPMOs generate oxidized cellulose fragments from plant cell wall material that can enhance immune responses compared with non-oxidized cellulose fragments, indicating that oxidized oligosaccharides can function in plant immune signaling (Zarattini et al. 2021). Oxidized products also arise during LPMO-mediated chitin degradation (Vaaje-Kolstad et al. 2010), and LPMO-generated oxidized chitin fragments have been reported to stimulate murine macrophages (Li et al. 2026). However, whether oxidized chitin fragments contribute to plant immune signaling remains unknown.

GH18 chitinases can fulfill analogous immune-suppressive functions. For example, *Mo*Chia1 is secreted into the rice apoplast by *Magnaporthe oryzae* during infection (Han et al. 2019, Yang et al. 2019). Resistant hosts employ a tetratricopeptide repeat protein (TPR) that inactivates *Mo*Chia1, thereby restoring immune responsiveness to fungal-derived chitin fragments. More broadly, degradation of fungal cell wall-derived immunogenic oligosaccharides by hydrolytic effector proteins is emerging as a common principle in immune evasion (Liu et al. 2023a) and fungal chitinases expand this effector repertoire (Fig. 2).

A central question in plant-microbe interactions is how host targeting effectors arise from existing microbial protein repertoires. Recent work has proposed an evolutionary link between antimicrobial effectors involved in microbial niche competition and effectors that modulate host plant immunity (Mesny et al. 2026). Given that some chitinases act in fungal antagonism whereas others suppress chitin-triggered immunity, microbial chitinases provide an interesting system in which to examine whether similar functional transitions can occur.

A recent example of chitinase functional diversification comes from the root endophyte *S. indica*. The antimicrobial GH18-CBM5 chitinase *Si*CHIT has a closely related paralog, *Si*CHIT2, located directly adjacent in the genome. The two chitinases are highly similar but structurally distinct, as *Si*CHIT2 lacks the C-terminal CBM5 domain. Whereas *Si*CHIT acts during fungal antagonism, the CBM5-lacking *Si*CHIT2 is expressed during host colonization and evades chitin-triggered immunity, thereby facilitating root colonization (Eichfeld et al. 2026). This functional difference appears to result from both transcriptional diversification and altered substrate preference: *Si*CHIT2 shows increased hydrolytic activity toward small, soluble immunogenic chitin oligomers compared with the CBM5-containing *Si*CHIT. Similar effects have been observed in other chitinases and may reflect interference of CBM5 domains with enzyme-substrate association or product dissociation on soluble oligomeric substrates, particularly under conditions of high substrate accessibility or saturation (Itoh et al. 2006, Uni et al. 2012). Thus, the *Si*CHIT/*Si*CHIT2 pair illustrates how changes in domain architecture, together with regulated expression, can shift chitinase function from fungal antagonism toward host immune modulation. Whether comparable domain changes have contributed to chitinase specialization in other fungal lineages remains to be tested.

Immune-suppressive chitinase variants can also arise through changes in the catalytic motif. The hemibiotrophic pathogens *Moniliophthora perniciosa* and *M. roreri* independently encode inactive GH18 chitinase-like effectors, in which the conserved residues of the DxxDxDxE motif are substituted, including replacement of the catalytic glutamate or aspartate by glutamine or asparagine, respectively. These chitinase-like effectors scavenge immunogenic COS during cacao infection and therefore function analogously to the LysM effector Ecp6 (de Jonge et al. 2010, Fiorin et al. 2018). The repeated emergence of such chitinase-like effectors in lineages with similar lifestyles suggests that structurally distinct solutions can converge on immune suppression. Thus, immune-suppressive chitinase functions are shaped not only by expression patterns, but also by structural changes that alter catalytic activity, substrate binding, or product turnover (Fig. 2). How additional amino acid variation in the substrate-binding cleft, including substitutions of aromatic residues that affect processivity and substrate preference (Horn et al. 2006, Zakariassen et al. 2009) influences ecological function, remains an open question. Together, these examples support the view that fungal GH18 chitinases are useful models for studying how conserved enzymes can specialize toward distinct ecological roles in pathogenic and mutualistic interactions.

## Beyond immune evasion: chitinases and symbiosis signaling?

Chitin-derived molecules not only activate plant immune responses but also function as symbiotic signals during the formation of arbuscular mycorrhizal (AM) symbioses (Maillet et al. 2011, Sun et al. 2015, Feng et al. 2019, Khokhani et al. 2021). For ectomycorrhizal fungi, such signals have so far only been reported for *Laccaria bicolor* (Cope et al. 2019). These so-called myc-factors activate host nuclear calcium oscillations upon perception, thereby initiating symbiosis formation (Khokhani et al. 2021, Crosino and Genre 2022). Chitin-based symbiotic signals comprise lipo-chitooligosaccharides (LCOs) and short-chain COS with a DP of 4–5. LCOs are short-chain COS substituted with a fatty acid at the non-reducing end, which distinguishes them from unsubstituted chitin-derived COS. Despite their structural similarity to immunogenic COS with DP ≥ 6, short-chain COS do not activate classical immune hallmarks but instead initiate symbiotic calcium oscillations (Genre et al. 2013).

Recent studies have begun to resolve how plant hosts assemble specific LysM receptor complexes to discriminate between immunogenic long-chain COS and symbiotic short-chain COS, thereby balancing immune and symbiotic signaling on the relative abundance of short-chain COS (Zhang et al. 2024, Tan et al. 2025). Strigolactones induce LCO and COS production (Genre et al. 2013, Tan et al. 2025), but the enzymatic mechanisms by which fungal symbionts generate these molecules remain unclear. Fungal chitinases have been proposed as possible contributors to this process (Khokhani et al. 2021, Crosino and Genre 2022) and transcriptome studies have reported induction of GH18 chitinases during symbiotic colonization (Martin et al. 2008, Tisserant et al. 2012). However, these observations have not yet been directly linked to LCO or COS production.

Exo-acting and many endo-acting GH18 chitinases produce chitobiose as the main reaction product. Because chitobiose is not an active symbiotic signal, chitinases would require atypical product profiles to contribute to the production of symbiotic COS, such as chitotetraose or to LCO biosynthesis (Fig. 2). Although reports of such chitinases are limited, the example of Chit33 from *T. harzianum* shows that endo-acting GH18 chitinases can produce chitotetraose as one of the main reaction products (Kidibule et al. 2020). The product spectra of GH18 chitinases from mycorrhizal fungi remain largely unexplored, but their characterization could reveal whether fungal symbionts enzymatically tune COS mixtures released into the apoplast during symbiosis establishment. Moreover, symbiotic COS and LCOs likely arise from coordinated activities of multiple chitin-modifying enzymes, including chitin synthases, chitin-deacetylases and potentially chitinases (Khokhani et al. 2021, Crosino and Genre 2022).

Resolving the molecular interplay among chitin-modifying enzymes and identifying the structural features that enable production of defined symbiotic signals could clarify whether chitinases contribute to mycorrhizal signaling. This would extend the ecological relevance of chitinases beyond antagonism and immune evasion, while also providing insights into the enzymatic basis of mycorrhizal symbiosis establishment.

## Conclusion and outlook

Microbial chitinases have been studied for decades and their roles in fungal physiology, nutrient acquisition, and fungal antagonism are well described. Here, we argue that these enzymes also contribute to plant-associated microbial community defense, niche establishment, and host colonization. In bacteria and fungi, chitinases can restrict fungal competitors, support access to chitin-derived nutrients, and, in some cases reduce chitin-triggered plant immunity by degrading or scavenging immunogenic chitin fragments. Thus, microbial chitinases represent one component of the broader microbial effector repertoire acting at microbial and host interfaces.

In ecological contexts, chitinases may help restrict fungal ingress into stable plant-associated communities. Conversely, pathogen-secreted chitinases could contribute to microbial community destabilization during host colonization, analogous to other antimicrobial effectors deployed by plant pathogens. However, this possibility remains largely unexplored and should be tested directly by linking chitinase activity to changes in microbiota or mycobiota composition during infection.

A central conclusion from recent studies is that chitinolytic activity alone does not explain the diverse biological roles of microbial chitinases. Instead, their functions emerge from the interplay between catalytic properties, structural features such as CBMs or loop regions, substrate accessibility, and regulated spatiotemporal expression. These functions are further shaped by synergistic activities between different microbial taxa, concerted transcriptional regulation with other CAZymes, and microbial metabolites. Variation in remote structural elements as well as amino acid substitutions within the catalytic cleft can alter substrate preference, processivity, product profiles, and biological activity. Understanding how these features map onto ecological context will be essential for explaining why closely related chitinases can act in fungal antagonism, immune evasion, or potentially symbiotic signaling.

Future studies should therefore move beyond measuring chitinolytic activity alone and combine structural, biochemical, genetic, and community-level approaches. Key priorities include defining how different CBM families affect chitinase function in fungal cell walls, identifying remote structural features that tune substrate engagement, determining whether pathogen chitinases reshape host-associated mycobiota, and testing whether chitinases contribute to the production of symbiotic chitin-derived signals. Extending this framework to other CAZyme families will further clarify how carbohydrate-active enzymes are adapted to specific ecological roles in the plant holobiont.

Although microbial chitinases are ancient and widespread enzymes, recent findings highlight their context-dependent activities at the intersection of microbial competition, host immune modulation, and community function. This makes them valuable model systems for studying how conserved enzymatic scaffolds become specialized molecular tools in complex plant-associated ecosystems.
